# A Robust Capillary Electrophoresis with Laser-Induced Fluorescence Detection (CE-LIF) Method for Quantitative Compositional Analysis of Trace Amino Acids in Hypersaline Samples

**DOI:** 10.1021/acsearthspacechem.3c00162

**Published:** 2023-10-19

**Authors:** K. Marshall Seaton, Chad I. Pozarycki, Nickie Nuñez, Amanda M. Stockton, The Oceans Across Space and Time team

**Affiliations:** † School of Chemistry & Biochemistry, Georgia Institute of Technology, Atlanta, Georgia 30332, United States

**Keywords:** astrobiology, brines, extraterrestrial analogues, planetary science, magnesium

## Abstract

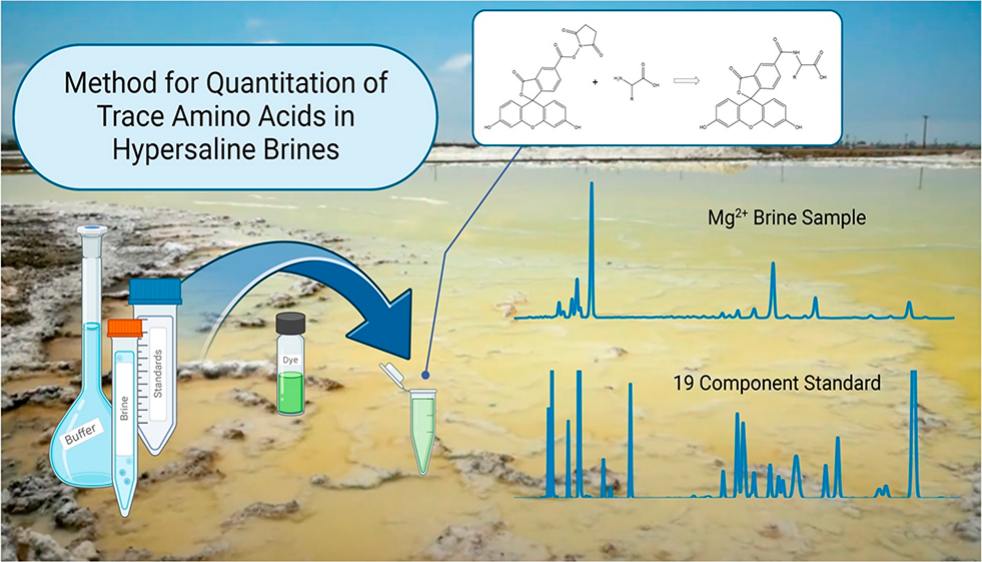

The search for life in our solar system can be enabled by the characterization of extreme environments representing conditions expected on other planets within our solar system. Molecular abundances observed in these environments help establish instrument design requirements, including limits of detection and pH/salt tolerance, and may be used for validation of proposed planetary science instrumentation. Here, we optimize capillary electrophoresis with laser-induced fluorescence detection (CE-LIF) separations for low limit of detection quantitative compositional analysis of amino acids in hypersaline samples using carboxyfluorescein succinimidyl ester (CFSE) as the amine-reactive fluorescent probe. Two methods were optimized for identification and quantification of proteinogenic amino acids, those with and those without acidic side chains, with limits of detection as low as 250 pM, improving on previous CFSE-amino acid CE-LIF methods by an order of magnitude. The resilience of the method to samples with high concentrations of Mg^2+^ (>4 M diluted to >0.4 M for analysis) is demonstrated on a sample collected from the salt harvesting facility South Bay Salt Works in San Diego, CA, demonstrating the highest Mg^2+^ tolerance for CE-LIF methods used in amino acid analyses to date. This advancement enables the rapid and robust analysis of trace amino acids and the search for biosignatures in hypersaline systems.

## Introduction

The relative abundance of individual amino acids within a sample provides insight into the chemical history of the sample.^1^ Abiotic processes, such as those contributing to the organic composition of meteoritic and cometary material, give rise to distributions of amino acids that are dominated by structurally simple, short-chained species. These typically have enantiomeric excesses that are quite low, while biotic processes give rise to amino acid distributions likely dictated by evolutionary selection rather than simply those that are kinetically or thermodynamically favored.^2,3^ Quantitative compositional analysis of the amino acids present is therefore a viable approach to assess the degree to which the organic content within a sample is of biotic or abiotic origin.

Examining patterns in the relative distributions of molecules within a sample provides a powerful and relatively agnostic approach for the determination of the chemical history of a planetary body including, perhaps most powerfully, our search for life beyond Earth.^4^ Among the many compound classes indicative of life, amino acids are ubiquitous in both terrestrial biology and extraterrestrial materials including meteorites and comet particles.^5,6^ Chiral amino acids can undergo enantiomeric enrichment, which, although not uniquely biogenic,^7^ is a defining characteristic of terrestrial biology, which further increases the utility of amino acids in associating the chemistry of a sample with a potentially biological origin.

Molecular abundances in extreme environments at the limits of life on Earth are often used to constrain the abundances of chemical biosignatures expected in extreme environments on other planetary bodies. Terrestrial analogues never completely replicate extraterrestrial environments, but they can mimic properties such as water activity, mineral matrices, low biomass, and other physicochemical properties expected in extreme planetary environments. Perhaps inconveniently, some of the most interesting and astrobiologically relevant environments in the solar system are often laden with salts. Among these, Jupiter’s icy moon Europa is believed to host a potentially habitable subsurface ocean, making it a compelling target in the search for life beyond Earth.^8^ Geologically young, nonicy material on Europa’s surface transported through solid-state convection expected to occur within Europa could allow access to subsurface ocean material through in situ compositional analysis;^9,10^ however, high concentrations of MgSO_4_ and/or H_2_SO_4_ expected in these regions could likely complicate the analysis of nonicy material.^11,12^ Studies of the Martian surface and atmosphere also indicate that Mars was likely much warmer and wetter in its distant past, possibly hosting conditions capable of sustaining life as we know it. Surface water on Mars is believed to have dried up billions of years ago, likely forming acidic hypersaline pools, which then may have deposited any organic content into salt evaporites.^13,14^ Methods utilized in studying environments with conditions similar to these systems (e.g., analogue environments on Earth) must therefore be highly sensitive and capable of detecting low-abundance species while also being robust to high salinities and complex ionic compositions.

Separation and detection methods traditionally used in amino acid analysis have historically been challenged by high-salt sample matrices. Gas chromatography–mass spectrometry (GC–MS) has been utilized as an in situ organic analysis technique for decades, most recently using chemical derivatization to enable the volatilization and detection of amino acids; however, this approach has been shown to complicate data analysis via undesirable contributions to the organic background signal.^15,16^ Liquid chromatography–mass spectrometry (LC–MS) has also been widely employed in the analysis of amino acids; however, high salt concentrations are incompatible with most LC–MS methods due to ion-source contamination and signal suppression.^17^ LC can be paired with ultraviolet–visible (UV–vis) detection to circumvent this issue; however, this results in drastically reduced sensitivity and undesirably high detection limits,^18^ prohibiting the detection and quantification of the low abundances of amino acids expected in energy limited systems on Earth or elsewhere. Thus, the need for sensitive amino acid analysis methods robust to high salt concentrations is clear.

Capillary electrophoresis (CE) is a powerful separation technique that offers exceptionally high separation efficiencies, extremely small sample volumes and reagent consumption, and can be coupled to numerous detection systems.^19^ When coupled with a laser-induced fluorescence (LIF) detection system, CE can provide sub-nM (or mere attomoles of molecules) limits of detection (LODs).^19−23^ Multiple amine-reactive fluorophores have been developed, enabling quantitative compositional analysis of ultralow abundance amino acids via CE-LIF.^24^ While sample salinity can lead to electro-dispersive effects that reduce resolution and signal, optimization of methodological parameters has been shown to be effective at minimizing the deleterious effects of high salinity.^20,23^ Multivalent cations are most problematic, as these can alter intercapillary surface chemistries, leading to electroosmotic flow (EOF) inhibition in addition to dispersive effects,^25^ resulting in drastically reduced peak resolution, sensitivity, and separation efficiency at cation concentrations as low as 5 mM.^20^ The addition of EDTA to the sample has been shown to mitigate these effects,^23^ but this adds an additional sample processing step. Here, we present a simple and robust CE-LIF amino acid analysis method that can be applied to samples with complex ionic compositions similar to those expected on other solar system bodies. We optimize the buffer separately for amino acids with acidic side chains from those with basic and neutral side chains. Separation figures of merit are determined, including LODs, for six amino acids. The optimized method is then used to analyze a hypersaline Mg^2+^-rich brine sample obtained from South Bay Salt Works (SBSW) in San Diego, California, demonstrating the suitability of this method in the analysis of complex brine systems.

## Experimental Section

### Chemicals and Reagents

All reagents were used as received, except where indicated. Sodium tetraborate decahydrate was purchased from Alfa Aesar (Tewksbury, MA). HCl, NaOH, and all amino acids were purchased from Sigma-Aldrich Co. (St. Louis, MO). Dimethylformamide (DMF) was purchased from Acros Organics (Geel, Belgium). Carboxyfluorescein succinimidyl ester (CFSE) was purchased from Life Technologies (Carlsbad, CA). Buffers, capillary conditioning solutions, and amino acid standards were prepared with water filtered to an 18 MΩ cm resistivity using a Barnstead GenPure UV-TOC/UF xCAD Ultrapure System (Lake Balboa, CA).

### CE-LIF Instrumentation

CE experiments were performed using a SciEx PACE MDQ system (Brea, CA) with laser-induced fluorescence detection at 488 nm excitation and an Omega Optical RapidBand Filter (Brattleboro, VT). Hydrodynamic injections were done at 0.4 psi for 5 s. Fused silica 60 μm inner-diameter capillaries were purchased from Polymicro Technologies (Phoenix, AZ). For all CE separations, capillaries were cut to 60 cm total length (50 cm effective length), and a 0.5 kV/cm separation potential was used. Each time a fresh capillary was cut, the capillary was conditioned for 15 min each with 0.1 M NaOH, water, 0.1 M HCl, water, and 40 mM sodium tetraborate (pH 9.2), followed by application of a 0.5 kV/cm potential across the capillary for 10 min for buffer equilibration. Prior to each separation, the capillary was rinsed with 0.1 M NaOH for 2 min and then rinsed for 4 min with the corresponding separation buffer.

### Data Processing

Data processing was performed using PeakFit version 4.12 (Systat Software, Inc., San Jose, CA) and OriginPro 2022 (OriginLab Co., Northampton, MA) software. Signal-to-noise ratios (*S*/*N*) were calculated as the ratio of peak amplitude to noise, where noise was calculated as the standard deviation of signal over a 2 min interval where no peaks were observed. All electropherograms were smoothed using a 0.1% Loess filter.

### Reagent Preparation

All buffer solutions were prepared from a 100 mM stock solution of sodium tetraborate (pH 9.2). For separation optimization experiments, all amino acid solutions were first prepared individually at 1 mM in 40 mM sodium tetraborate. To prepare the amino acid separation optimization standard, 1 mL each of 1 mM alanine (Ala), arginine (Arg), asparagine (Asn), aspartic acid (Asp), glutamic acid (Glu), glutamine (Gln), glycine (Gly), histidine (His), isoleucine (Ile), leucine (Leu), lysine (Lys), methionine (Met), phenylalanine (Phe), proline (Pro), serine (Ser), threonine (Thr), tryptophan (Trp), tyrosine (Tyr), and valine (Val) in 40 mM sodium tetraborate were added individually to a 50 mL Falcon tube and diluted to 40 mL with 40 mM sodium tetraborate, resulting in a final concentration of 25 μM for each amino acid. A 2 mM solution of CFSE dissolved in DMF was prepared for amino acid labeling and stored at −20 °C when not in use. Separation optimization standards were labeled by combining 1 μL of 2 mM CFSE solution with 99 μL of an amino acid standard and allowed to react in the dark for 1 h prior to analysis.

For LOD measurements, buffer solutions were prepared in a HEPA-filtered organic cleanroom using sterile procedures. All water used in LOD experiments was triply distilled prior to use, and all sodium tetraborate was recrystallized five times before use to reduce organic contamination. After distillation and recrystallization, water and sodium tetraborate were exposed to UV irradiation for 24 h to degrade any background amino acids present. To prepare the neutral and basic amino acid LOD standard, 1 mL each of 1 mM Gly, Leu, Met, Pro, and Ser in 70 mM sodium tetraborate was added to a 10 mL Falcon tube, then diluted to a volume of 10 mL, resulting in a final concentration of 100 μM for each amino acid. The acidic amino acid LOD standard was prepared in the same manner as the neutral and basic amino acid LOD standard by using only Asp. Neutral and basic amino acid standards were diluted to 1 μM, 500 nM, 200 nM, 100 nM, 50 nM, 20 nM, 10 nM, 5 nM, 2 nM, and 1 nM with 70 mM sodium tetraborate and then individually labeled at each concentration by adding 1 μL of CFSE solution to 99 μL of standard and allowed to react overnight. Acidic amino acid standards were diluted to 10 μM, 5 μM, 2 μM, 1 μM, 500 nM, 200 nM, 100 nM, 50 nM, 20 nM, and 10 nM with 30 mM sodium tetraborate and then labeled in the same manner as the neutral and basic amino acids. After derivatization, the standards were analyzed without further dilution.

### Real Sample Collection, Prior Characterization, and Preparation

The brine sample analyzed in this work was collected from a salt evaporation pond on August 6, 2019, at SBSW in San Diego, California. The sample was collected in a sterile 50 mL polypropylene centrifuge tube using a standoff pumping system where a 2 m pole with plastic tubing was placed into the brine ∼30 cm from the surface and away from the edge of the brine pool and then peristaltically pumped into the centrifuge tube. Measurements performed at the sample collection site or soon after collection show a brine water activity (*a*_w_) of 0.39, pH of 5.30, [Na^+^] of 0.14 M, [Mg^2+^] of 4.20 M, [Cl^–^] of 7.56 M, [SO_4_^2–^] of 0.23 M, and 403 g/L of total dissolved solids.^26^ Due to concurrent osmotic and chaotropic stress, the MgCl_2_-saturated brine sample used in this work is presumed to be devoid of active life, as the measured water activity is well outside accepted limits for metabolic activity and cellular division. Prior to the amino acid analysis conducted here, brine samples were taken from storage at room temperature and vortexed for 5 s. After settling for 5 min, 1 mL aliquots were taken and centrifuged at 2000 rpm for 5 min. A fraction of the supernatant (0.5 mL) was removed and diluted five times with 2 mL of the corresponding running buffer. Samples and blanks were prepared for analysis via standard addition by combining with running buffer, CFSE, and an amino acid standard. After preparation, the sample (or blank) is diluted to a factor of 10, with a CFSE and sodium tetraborate concentration of 20 μM and ∼62.5 mM, respectively. Samples were allowed to react in the dark at room temperature for 24 h prior to analysis without further dilution.

## Results and Discussion

### Amino Acid Separation

**Figure 1:**
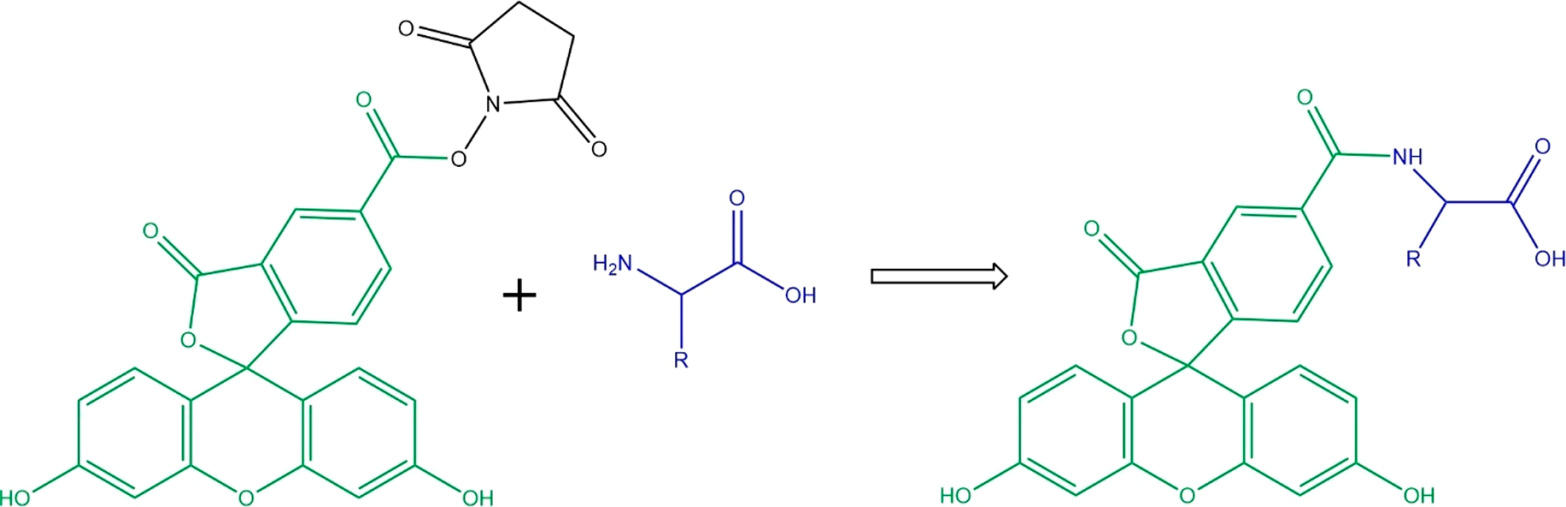
Fluorescent labeling reaction between an amino acid and carboxyfluorescein succinimidyl ester (CFSE).

Nineteen amino acids (Ala, Arg, Asn, Asp, Glu, Gln, Gly, His, Ile, Leu, Lys, Met, Phe, Pro, Ser, Thr, Trp, Tyr, and Val) were used in the separation optimization standard. CFSE was selected for fluorescent derivatization (Figure 1) due to the rapid reactivity of the succinimidyl ester moiety toward primary amines, its superior extinction coefficient and quantum yield at 488 nm, minimal fluorescent dye side products, and exceptionally low LODs when used to derivatize amino acids.^3,27^

**Figure 2:**
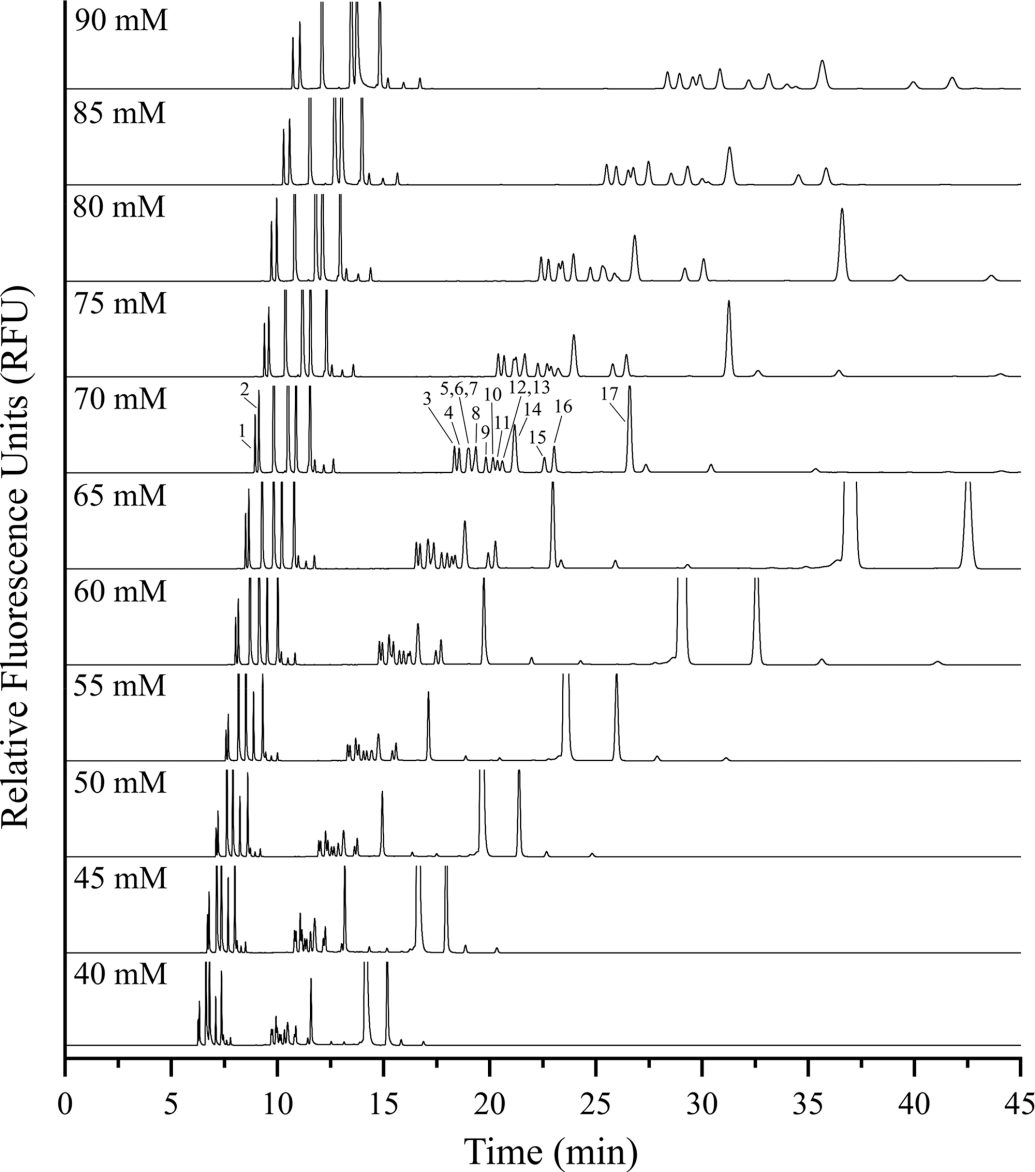
Separation optimization of neutral and basic amino acids through variation of the sodium tetraborate concentration in the running buffer. The amino acid standard includes (1) Arg, (2) Lys, (3) Leu, (4) Ile, (5) His, (6) Tyr, (7) Gln, (8) Met, (9) Val, (10) Phe, (11) Asn, (12) Thr, (13) Trp, (14) Pro, (15) Ser, (16) Ala, and (17) Gly, each at a concentration of 25 μM. Buffer pH is 9.2, and separations are conducted at 30 kV with a 60 cm total capillary length (50 cm effective length).

**Figure 3:**
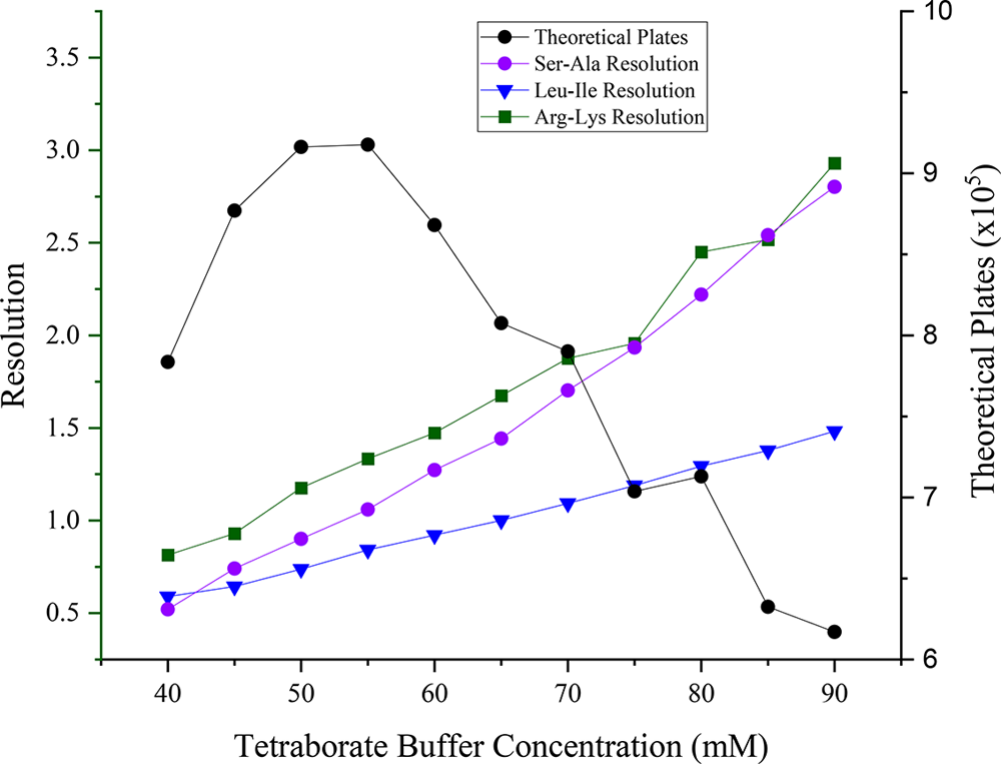
Resolution of the Ser–Ala, Leu–Ile, and Arg–Lys peak pairs and peak efficiency in theoretical plates plotted as a function of sodium tetraborate concentration in the running buffer. Data are extracted from the electropherograms shown in Figure 2.

**Figure 4:**
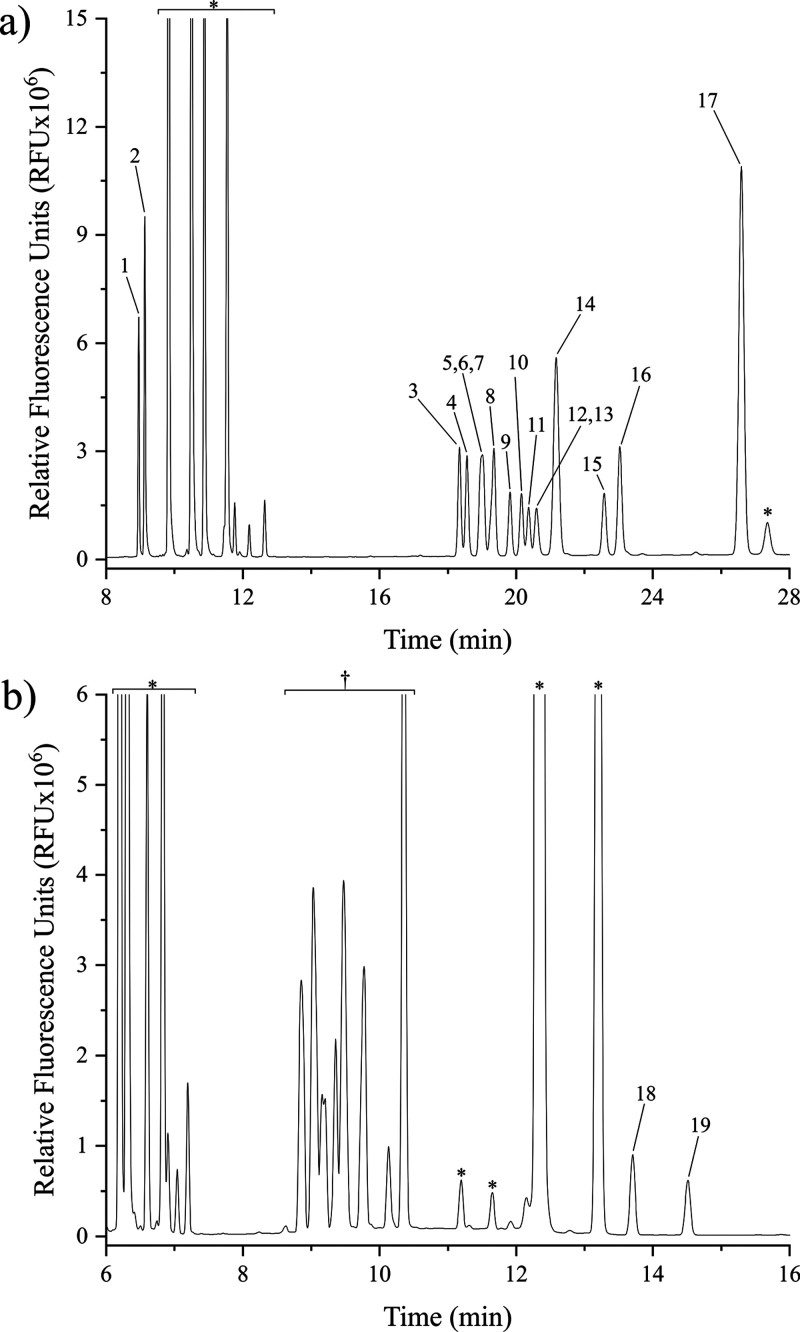
Optimized separations of (a) neutral and basic CFSE-labeled amino acids in 70 mM sodium tetraborate; and (b) acidic CFSE-labeled amino acids in 30 mM sodium tetraborate. Peaks: Arg (1), Lys (2), Leu (3), Ile (4), His (5), Tyr (6), Gln (7), Met (8), Val (9), Phe (10), Asn (11), Thr (12), Trp (13), Pro (14), Ser (15), Ala (16), Gly (17), Glu (18), and Asp (19). Buffer pH is 9.2, and separations are conducted at 30 kV with a 60 cm total capillary length (50 cm effective length). ^†^Unresolved neutral and basic amino acids. *Dye side products.

Separations of the amino acid standard were conducted with sodium tetraborate concentrations in the running buffer that systematically varied between 40 and 90 mM in 5 mM increments (Figure 2). Increasing buffer concentration (and therefore the ionic strength of the solution) results in a reduction in EOF, which lengthens analysis times and therefore provides increased peak resolution. However, a trade-off exists between peak resolution, peak efficiency, and analysis times, so an empirical evaluation of each of these figures of merit is often necessary to determine optimal separation conditions. Using data from these experiments, both the sum of theoretical plates and resolution for the Ser-Ala, Leu-Ile, and Arg-Lys peak pairs were calculated to examine peak efficiency and resolution as a function of buffer concentration (Figure 3). Although higher sodium tetraborate concentrations continued to increase peak resolution for most peak pairs up to 90 mM, substantially longer analysis times were observed with increasing concentration beyond 60 mM. The sum of theoretical peaks for the three peak pairs increased from 40 to 55 mM, and then began to decrease after 60 mM, exhibiting a sharp decline in peak efficiency observed after 70 mM. From these data, 70 mM was chosen as the optimal buffer concentration to use for further separations of neutral and basic amino acids, in which 12 of the 17 neutral and basic amino acid components of the standard (Arg, Lys, Leu, Ile, Met, Val, Phe, Asn, Pro, Ser, Ala, and Gly) were resolved simultaneously (Figure 4a). His, Tyr, and Gln comigrated, as did Thr and Trp. Migration times, peak efficiencies, peak resolutions (*R*_s_), peak areas, and *S*/*N* for the optimized neutral and basic amino acid separation are provided in Table 1. Peak resolutions of 0.9 or better were obtained for most amino acids in the separation standard. Resolutions of 0.5 or greater enable peak identification and estimations of quantity; however, it is widely accepted that *R*_s_ values approaching unity or greater yield the most accurate quantitation capability. We note that all native organic compounds with a primary amine group will be labeled and separated using this method; however, a full characterization of all unidentified peaks is beyond the scope of this manuscript and is planned for follow-up work.

**Table 1:** Migration Times, Peak Efficiencies, and Resolutions for Analyte Peaks in Neutral and Basic Amino Acid Separation Are Shown in Figure 4a^a^

Amino Acid Peak	Migration Time (min)	Peak Efficiency (plates/m)	Resolution (*R*_s_)	Peak Area	Signal-to-Noise Ratio
Arg	8.95	330000		2.9 × 10^5^	5.2 × 10^3^
Lys	9.13	290000	1.9	4.4 × 10^5^	7.3 × 10^3^
Leu	18.34	310000		3.0 × 10^5^	2.5 × 10^3^
Ile	18.57	320000	1.2	2.7 × 10^5^	2.3 × 10^3^
His/Tyr/Gln	19.00	130000	1.8	4.4 × 10^5^	2.3 × 10^3^
Met	19.35	230000	1.3	3.6 × 10^5^	2.5 × 10^3^
Val	19.82	250000	2.1	2.1 × 10^5^	1.5 × 10^3^
Phe	20.16	260000	1.5	2.0 × 10^5^	1.5 × 10^3^
Asn	20.38	270000	1.0	1.6 × 10^5^	1.1 × 10^3^
Thr/Trp	20.59	200000	0.9	1.8 × 10^5^	1.1 × 10^3^
Pro	21.17	120000	1.9	1.0 × 10^6^	4.6 × 10^3^
Ser	22.58	220000	4.5	2.4 × 10^5^	1.4 × 10^3^
Ala	23.04	230000	1.7	4.3 × 10^5^	2.5 × 10^3^
Gly	26.59	210000	11.8	1.9 × 10^6^	9.0 × 10^3^

a Resolution for each amino acid peak is calculated with respect to the previously eluted amino acid peak in the standard. Because of dye peaks eluting between Leu and Lys, there is not a resolution calculated between these peaks.

Simultaneously resolving the maximum number of neutral and basic amino acids required the use of relatively high buffer concentrations, which resulted in undesirable elution times for Glu and Asp under these conditions (>60 min). Although Glu and Asp could, in principle, be detected and quantified within the same run as the neutral and basic amino acids, the conditions used for the aforementioned separation would result in long (>1 h) analysis times and drastically reduced peak intensities and efficiencies for these species. Because of this, a separate method for their separation and quantification was implemented. Using the same standard, sodium tetraborate concentrations were varied from 10 to 35 mM in 5 mM increments to identify optimal separation conditions for the acidic amino acids (Supplementary Figure S1). Using a running buffer consisting of 20 mM sodium tetraborate, Asp and Glu were baseline resolved from both each other and neighboring dye peaks in ∼10 min; however, upon analyzing samples with a low ratio of analyte to fluorescent dye, the neighboring dye peak was found to partially eclipse the Glu peak under these conditions. To avoid this, 30 mM sodium tetraborate was used for the analysis of acidic amino acids such that both were detectable in the presence of excess fluorescent dye (Figure 4b).

### Limits of Detection

**Figure 5:**
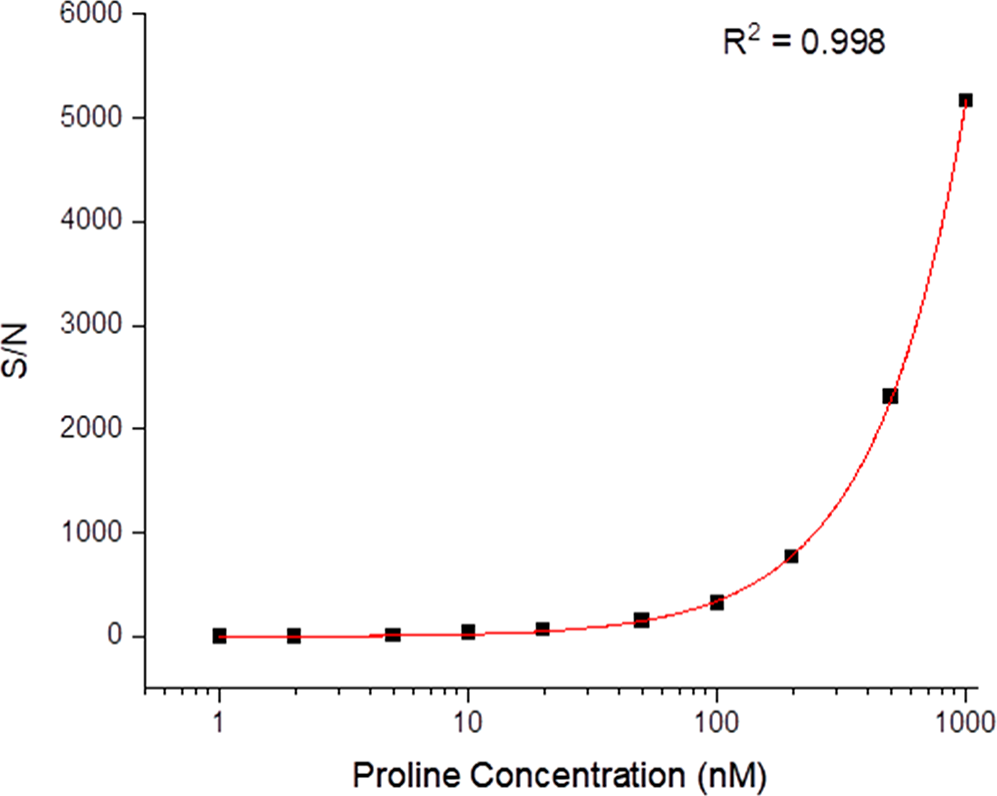
An example limit of detection (LOD) plot of one of three triplicate analyses fitted to a power law. Separations were conducted in 70 mM sodium tetraborate, pH 9.2, at 30 kV with a 60 cm total capillary length (50 cm effective length).

The LOD of this method was assessed using Leu, Met, Pro, Ser, Gly, and Asp, as this suite represents multiple amino acids with varying side chain functionalities and labeling efficiencies that represent the range of analyte electrophoretic mobilities observed. Molar LODs for each amino acid were calculated from an average of triplicate experiments by the extrapolation of a power law fit to a *S*/*N* of 3 (Figure 5) using measurements made from amino acid concentrations ranging from 1 μM to 1 nM for neutral and basic amino acids and from 10 μM to 10 nM for acidic amino acids (concentrations prior to labeling). Despite recrystallizing sodium tetraborate several times, distilling purified water three times prior to buffer preparation, and irradiating both with UV light in a clean room to remove amino acid contamination, due to the superior sensitivity of the method, a small amount of Gly and Ser was observed in the procedural blank. Experiments to isolate the source of this contamination revealed that although buffer recrystallization and UV irradiation/distillation of water reduced the level of background amino acids present in the procedural blank, the dye itself is likely the cause of the remaining contamination. Due to the high cost and very low quantities of the dye available, further purification of the dye was not possible for this study but could be conducted when the need for a lower background justifies the time and monetary cost. To account for this, peak areas from 1, 2, 5, 10, and 20 nM concentrations were used to calculate concentrations of glycine and serine present in the blank via standard addition. For these species, the *S*/*N* was plotted vs the sum of the calculated contamination and the added concentration of Ser and Gly, respectively, to correct for this contamination. The calculated LODs for each species are presented in Table 2.

**Table 2:** Calculated Limits of Detection for Leu, Met, Pro, Ser, Gly, and Asp

Leucine	Methionine	Proline	Serine^a^	Glycine^a^	Aspartic Acid
380 ± 60 pM	1.1 ± 0.2 nM	800 ± 200 pM	1.2 ± 0.5 nM	250 ± 90 pM	4.6 ± 1.0 nM

a Values were corrected for contamination. Background levels of Ser and Gly were calculated to be 8.4 ± 4.0 nM and 4.8 ± 2.3 nM, respectively.

This method represents an improvement in LODs for CFSE-labeled Leu, Met, Pro, Ser, and Gly by roughly an order of magnitude relative to previous studies,^3,21,28^ representing the lowest detection limits reported for CFSE-labeled amino acids using capillary zone electrophoresis (CZE) to date. The LODs reported here (with the exception of Asp) are consistent with amino acid labeling efficiencies, which are dependent upon both side chain functionality and steric hindrance.

### South Bay Salt Works Sample Analysis

To explore the applicability of this method toward native hypersaline environments, a complex brine sample collected from SBSW, a salt harvesting facility in San Diego, California, was analyzed. SBSW serves as an analogue for Martian brines in ancient salt lake beds,^26,29^ as the sequential evapoconcentration of seawater here mimics the evaporation of ancient Martian lakes, which formed the salt deposits observed in shallow depressions on Mars today.^30,31^ Nonicy material on the surface of Europa is expected to harbor extremely high concentrations of magnesium as well.^11,32,33^

**Figure 6:**
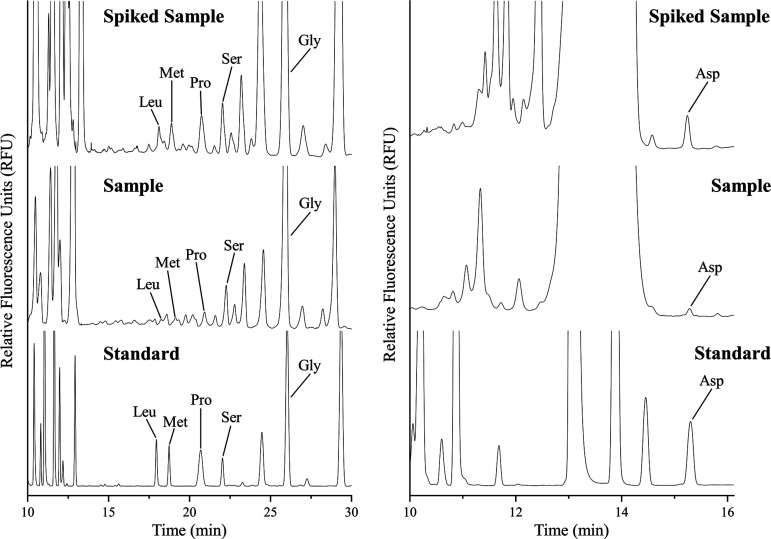
Analysis of a sample taken from South Bay Salt Works (SBSW), a salt harvesting facility, in San Diego, California. “Spiked Sample” represents a 250 (left) and 600 (right) nM standard addition of the amino acids shown in the electropherogram. Separations were conducted in 70 mM sodium tetraborate (left) and 30 mM sodium tetraborate (right), pH 9.2, at 30 kV with a 60 cm total capillary length (50 cm effective length).

CZE analysis by standard addition was performed on the brine sample collected from SBSW after dilution by a factor of 10 in the corresponding running buffer (Figure 6). The results of these analyses are summarized in Table 3. To correct for any contamination present, a procedural blank was analyzed in triplicate and subtracted from the values obtained from the analysis of SBSW samples. Leu, Pro, Ser, Gly, and Asp native to the sample were quantified via standard addition at μM concentrations. Arg, Lys, Leu, Ile, His/Tyr/Gln, Met, Val, Phe, Asp, Thr/Trp, and Ala peaks were identified by spiking the sample with the 19-component standard and then the migration times of each amino acid in the native sample were compared with those obtained from spiking experiments, with estimated concentrations ranging from 70 ± 8 to 1500 ± 200 nM. Met was observed but below the limit of quantitation. These concentrations were estimated using matrix-corrected calibration curves generated from the standard additions of Leu, Pro, Ser, Gly, and Asp, which were then applied to the other amino acids in the standard based on similarities in migration times and peak area responses with respect to concentration. A detailed quantitative determination of the full suite of amino acids in SBSW samples of varying ionic compositions is underway and will be included in a future publication.

**Table 3:** Amino Acid Content in a Mg^2+^ Brine Sample Retrieved from the South Bay Salt Works (SBSW) via Standard Addition^a^

Leucine	Methionine	Proline	Serine	Glycine	Aspartic Acid
0.5 ± 0.3 μM	NQ^b^	0.7 ± 0.2 μM	1.4 ± 0.4 μM	2.4 ± 0.3 μM	1.5 ± 0.4 μM

a Error was calculated from the standard deviation of triplicate analyses.

b Not detected at quantifiable amounts.

Despite the high salt concentrations present in the sample after dilution (0.42 M Mg^2+^, 0.14 M Na^+^, 0.76 M Cl^–^, etc.),^26^ analyses showed only a small increase in noise levels over that of the procedural blank, demonstrating a high tolerance of this method toward saline matrices. The increased tolerance to Mg^2+^ reported here relative to that of previous CE-LIF methods is partially due to a high buffer concentration and thus ionic strength, which reduces dispersive effects and therefore mitigates the magnitude of EOF inhibition due to high salinity.^34^ This method is also inherently less prone to electrodispersive effects, as it uses hydrodynamic injection as opposed to electrokinetic injection (see Supporting Information). Although previous CE-LIF analyses have reported a significant deterioration in signal and peak resolution at <5 mM Mg^2+^,^20^ we demonstrate that samples with Mg^2+^ concentrations of >400 mM can be successfully analyzed for amino acid content using a simple running buffer consisting of only sodium tetraborate.

## Conclusions

The method presented here significantly improves on previous methods employed in the analysis of amino acids in Mg^2+^ brines, which have been shown to be prohibitive toward CE-LIF analysis of these species, using a simple running buffer consisting of only sodium tetraborate. Peak resolutions of >0.94 are achieved for neutral and basic amino acids, with 12 of the 17 neutral and basic amino acids in the standard resolved simultaneously in a single run using a running buffer consisting of only 70 mM sodium tetraborate. Analysis of acidic amino acids was carried out in a separate run using 30 mM tetraborate. In addition to a demonstrably higher tolerance toward high Mg^2+^ concentrations, we report an order-of-magnitude improvement in LODs for CFSE-labeled amino acids analyzed using CZE, as low as 250 pM for Gly. This method will further enable the full characterization of complex Mg^2+^-rich hypersaline systems representing the environments expected in other ocean worlds in our solar system.

## Data Availability

Data will be made available upon request.

## Supporting Information Available

Peak resolution for peak pairs in optimized neutral and basic amino acid separation; buffer optimization for acidic amino acid separation; acidic amino acid peak pair resolutions as a function of buffer concentration; figures of merit for optimized acidic amino acid separation; explanation of electrokinetic and hydrodynamic injections (PDF)

## Abbreviations

CE: capillary electrophoresis
LIF: laser-induced fluorescence
CFSE: carboxyfluorescein succinimidyl ester
GC–MS: gas chromatography–mass spectrometry
LC–MS: liquid chromatography–mass spectrometry
UV–vis: ultraviolet–visible
LOD: limit of detection
EOF: electroosmotic flow
DMF: dimethylformamide
*a*_w_: water activity
*R*_s_: peak resolution
*S*/*N*: signal-to-noise ratio
CZE: capillary zone electrophoresis
SBSW: South Bay Salt Works
