# Supplementary material for: A Robust Capillary Electrophoresis with Laser-Induced Fluorescence Detection (CE-LIF) Method for Quantitative Compositional Analysis of Trace Amino Acids in Hypersaline Samples
Source: ACS Earth Space Chem. 2023 Oct 19;7(11):2214–21. doi: 10.1021/acsearthspacechem.3c00162 (PMC10658621; doi:10.1021/acsearthspacechem.3c00162)
Supplement: Supplementary file 1 — sp3c00162_si_001.pdf [file sp3c00162_si_001.pdf]

A Robust Capillary Electrophoresis with Laser-Induced Fluorescence Detection (CE-LIF) Method for Quantitative Compositional Analysis of Trace Amino Acids in Hypersaline Samples

K. Marshall Seaton,[1] Chad I. Pozarycki,[1] Nickie Nuñez,[1] Amanda M. Stockton,[1*] and the Oceans Across Space and Time team[**]

1. School of Chemistry & Biochemistry, Georgia Institute of Technology, Atlanta, Georgia 30332, United States

[*]Corresponding author: amanda.stockton@chemistry.gatech.edu

[**]Oceans Across Space and Time field team for 2019 SBSW sample collection includes Britney E. Schmidt, Jeff S. Bowman, Jennifer B. Glass, Christopher E. Carr, Carlie Novak.

Supporting Information

Table S1. Neutral and basic amino acid peak pair resolution vs. sodium tetraborate concentration in the running buffer for separations shown in Figure 2.

	**Sodium Tetraborate Concentration (mM)**										
Amino Acid Peak Pair	**40**	**45**	**50**	**55**	**60**	**65**	**70**	**75**	**80**	**85**	**90**
1,2	0.81	0.96	1.07	1.35	1.48	1.71	1.93	2.16	2.51	2.53	3.11
2,3	40.19	42.57	48.23	55.57	55.1	59.87	65.21	62.51	69.69	64.03	69.64
3,4	0.54	0.64	0.73	0.84	0.91	1.00	1.20	1.15	1.32	1.32	1.44
4,5	1.59	1.75	1.85	1.67	1.78	1.68	1.77	1.74	1.75	1.65	1.60
5,6	0.60	0.71	0.82	0.89	1.12	1.19	1.32	0.51	0.61	0.70	0.84
6,7	0.95	1.17	1.30	1.55	1.71	1.89	2.08	1.61	1.75	2.02	2.30
7,8	0.67	0.76	0.90	1.05	1.17	1.31	1.49	2.33	2.55	2.8	3.16
8,9	1.44	1.44	1.38	1.36	1.25	1.07	0.95	1.66	1.73	1.95	2.03
9,10	1.03	1.17	1.36	0.41	0.57	0.76	0.94	0.69	0.44	1.64	1.74
10,11	2.18	2.4	2.93	1.57	1.68	1.80	1.87	1.02	1.32	2.60	2.84
11,12	0.59	0.70	0.90	3.30	3.58	4.09	4.49	1.89	2.23	6.07	6.62
12,13	6.36	6.98	7.66	1.07	1.23	1.48	1.68	4.98	5.32	2.52	2.87
13,14				8.56	9.67	10.77	11.79	2.00	2.20		
14,15								12.92	13.77		

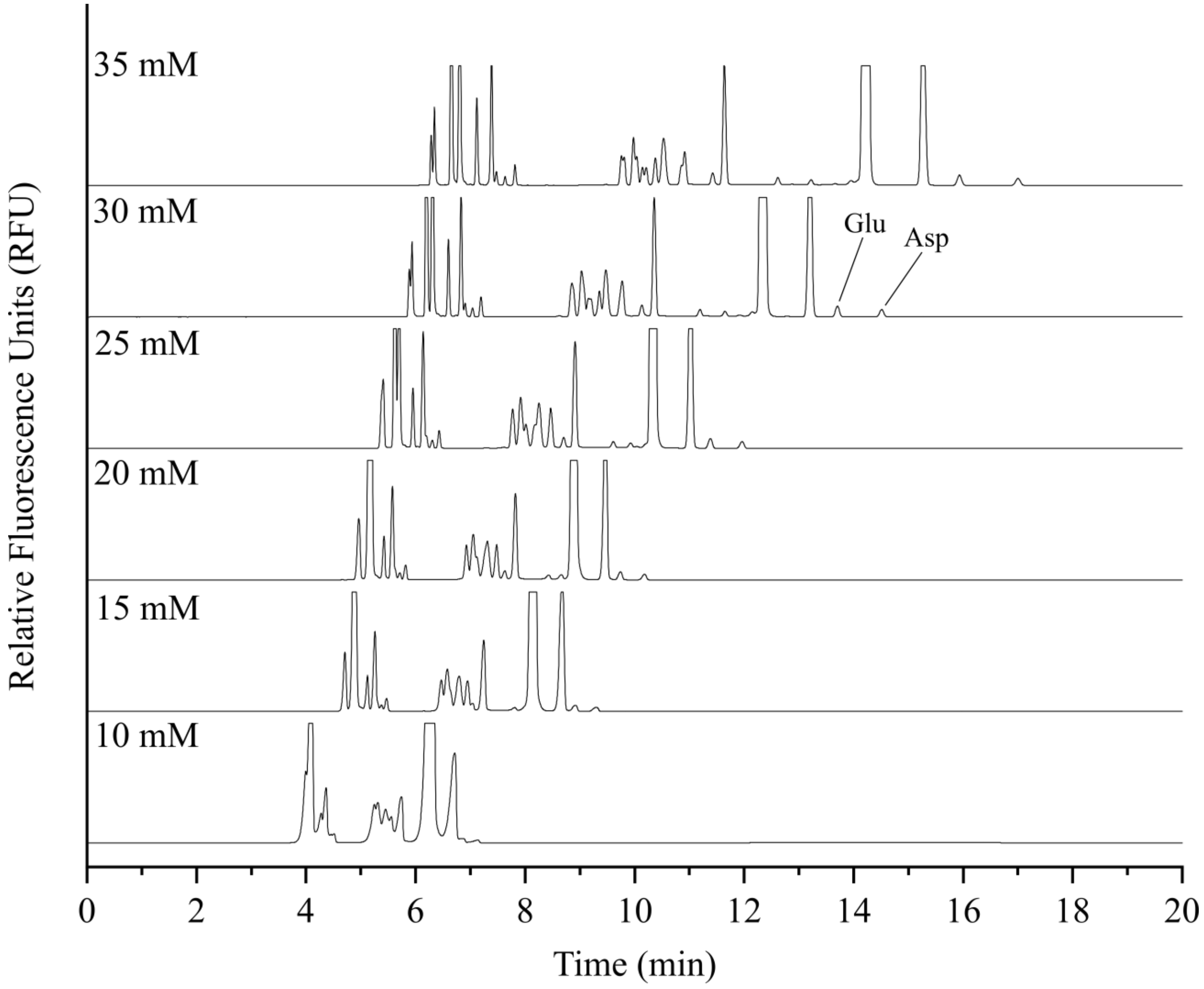

Figure S1. Separation optimization of acidic amino acids through variation of sodium tetraborate concentration in the running buffer. The amino acid standard includes glutamic and aspartic acid at a concentration of 25 µM. Buffer pH is 9.2, and separations are conducted at 30 kV with a 60 cm total capillary length (50 cm effective length).

Table S2. Acidic amino acid peak pair resolution as a function of sodium tetraborate concentration in the running buffer for separations shown in Figure S1.

	Sodium Tetraborate Concentration (mM)					
**Amino Acid Peak Pair**	**10**	**15**	**20**	**25**	**30**	**35**
Glu, Asp	0.53	1.89	2.69	3.43	4.84	5.9

Table S3. Migration time, peak efficiency, and resolution for amino acids comprising the acidic amino acid separation shown in Figure S1 for 30 mM sodium tetraborate.

Amino Acid Peak	Migration Time (min)	Peak Efficiency (plates/m)	R_s
Glu	13.70	113028	-
Asp	14.51	115879	4.84

Electrokinetic and Hydrodynamic Injection. Sample injection in capillary electrophoresis can be done in two ways, either electrokinetically (which uses an applied voltage to inject analytes based on their mobilities) or hydrodynamically (which uses a pressure differential as a means of sample injection). There are benefits and drawbacks to using either injection method; hydrodynamic injection is less discriminatory towards differences in analyte electrophoretic mobilities, while electrokinetic injection is beneficial for more viscous solvents and allows for chemical selectivity during sample injection. In the context of this work specifically, using electrokinetic injection for samples with different ionic strengths (blanks, standards, and real samples) would result in variable field strengths during injection, which would therefore result in different amounts of analyte being injected in samples with different chemical matrices. Hydrodynamic injection is less discriminatory towards samples with different ionic compositions, making it preferential for use in the analysis of hypersaline samples.
